# Calculation of Temperature Fields in a Lithium Ceramic Pebble Bed during Reactor Irradiation in a Vacuum

**DOI:** 10.3390/ma16216914

**Published:** 2023-10-27

**Authors:** Yevgen Chikhray, Timur Kulsartov, Zhanna Zaurbekova, Inesh Kenzhina, Kuanysh Samarkhanov

**Affiliations:** 1 Institute of Applied Sciences and Information Technologies, Almaty 050032, Kazakhstan; chikhray@physics.kz (Y.C.); tima@physics.kz (T.K.); kenzhina@physics.kz (I.K.); 2 Institute of Atomic Energy, Branch of National Nuclear Center, Kurchatov 071100, Kazakhstan

**Keywords:** lithium ceramics, pebble bed, temperature distribution, neutron irradiation, tritium

## Abstract

Two-phase lithium ceramic Li_2_TiO_3_-Li_4_SiO_4_ is considered as a tritium multiplier for use in the solid blanket of fusion reactors. To date, the most accurate understanding of the processes of tritium and helium production and release occurring in the breeder blanket materials under neutron irradiation can only be obtained from experiments in fission research reactors. At that, irradiations in vacuum give the possibility to register even very fast gas release processes (bursts) from the ceramics’ voids and pores, although it reduces the thermal conductivity of the pebble bed. The purpose of this work was to simulate the heating of mono-sized pebble bed (1 mm in diameter) of two-phase lithium ceramic 25 mol%Li_2_TiO_3_+75 mol%Li_4_SiO_4_ in an ampoule device during neutron irradiation at the WWR-K research reactor under vacuum conditions, and to determine experimental parameters in order to prevent heating of the lithium ceramics up to the Li_4_SiO_4_-Li_2_SiO_3_ phase transition temperatures (>900 °C). For the first time, it was obtained that the effective thermal conductivity of a 1 mm mono-sized pebble bed of 25 mol%Li_2_TiO_3_+75 mol%Li_4_SiO_4_ significantly decreases (four times) when it is irradiated with neutrons in a vacuum (at a helium pressure of approximately 10 Pa), compared to a similar calculation at 100 kPa of helium (when the He sweep is used). It was concluded that it is difficult to evaluate the maximal temperature of the ceramics in the capsule by measuring the temperature of its outer metal wall (according to thermocouple readings) without using the results of thermophysical calculations for each type of ceramic, taking into account its quantity, specific heat release and pebble size(s). To control the temperature of the ceramics during an irradiation experiment in a vacuum, an in-capsule thermocouple should be used, placed in the center of the pebble bed. Measuring the temperature of the pebble bed based on the capsule wall temperature can lead to overheating of the ceramics and phase changes.

## 1. Introduction

It is likely that tritium will be the main source of energy and fuel for future fusion reactors [1]. Unfortunately, there are no natural reserves of tritium in a stable form and therefore, it must be produced through nuclear reactions with lithium nuclei. The current approach to fusion reactor design is to use lithium-containing ceramics in the design of the breeder blanket that surrounds the fusion plasma. Such ceramics have proven themselves well in numerous experiments in fission reactors, demonstrating high thermal stability and good tritium yield parameters.

The primary functions of the tritium breeder blanket in a fusion reactor are to convert the kinetic energy of the fusion neutrons into recoverable heat and to ensure adequate tritium breeding to meet tritium fuel requirements throughout the life of the fusion reactor.

For the prospective fusion reactor design, it is desirable to maximize the recoverable heat produced in the blanket, which is defined as the energy released in the first wall, breeder, reflector and chamber per fusion neutron. Another important function of the blanket is that it acts as part of the three-dimensional reactor screen. While the blanket does serve many functions, the critical feature is the recovery of the generated tritium so that it can be used as fuel to keep the reactor running.

Radiation and heat resistance, neutron capture cross section and chemical stability have led to the widespread use of ceramic materials in the nuclear and thermonuclear industry. Ceramics are used to make fission reactor fuel (UO_2_ and ZrO_2_) and to shield and insulate materials of fission and fusion reactors, neutron absorbers, etc. [2,3]. Several oxides, in particular MgO, Al_2_O_3_ and their equimolar mixture MgAl_2_O_4_ single crystals and transparent ceramics have been considered as attractive candidates for diagnostics/optical windows in future fusion devices [4,5,6].

Lithium ceramics (Li_2_O, Li_2_ZrO_3_, Li_2_TiO_3_ and Li_4_SiO_4_) are considered as tritium multipliers (according to the ^6^Li(n,He)T nuclear reaction) for use in the solid blanket of fusion reactors [7,8,9]. The two-phase lithium ceramic Li_2_TiO_3_-Li_4_SiO_4_ combines the chemical stability of Li_2_TiO_3_ and the high density of lithium in Li_4_SiO_4_ [8,10,11]. Studies of lithium silicates (Li_4_SiO_4_ and Li_2_SiO_3_) have shown that these materials have good tritium solubility, they are compatible with structural materials and have sufficient thermophysical, chemical and mechanical stability at high temperatures. To load into the blanket, lithium ceramics are manufactured in the form of pebbles with a diameter of 0.1 to 3 mm. 

The most accurate understanding of the processes of tritium and helium production and release occurring in the breeder blanket materials can only be obtained from the results of irradiation experiments at the research nuclear reactors. However, irradiation by neutrons, electrons and heavy swift ions of ceramic oxides can cause induced activation, formation of gas bubbles, reduction in thermal conductivity, embrittlement and other material-degrading effects [12,13,14]. 

Previously, lithium ceramics were irradiated both in the in-situ mode with simultaneous measurement of the levels of evolved gases [15,16,17,18,19] and in the passive mode. The post-irradiation tritium release from Li_2_TiO_3_-Li_4_SiO_4_ two-phase ceramics has been studied in previous TDS studies [20,21,22,23,24]. Experimental results show that the intensity of the main tritium release peak during thermal desorption is controlled by the ratio of the Li_2_TiO_3_ and Li_4_SiO_4_ phases. 

**Figure 1:**
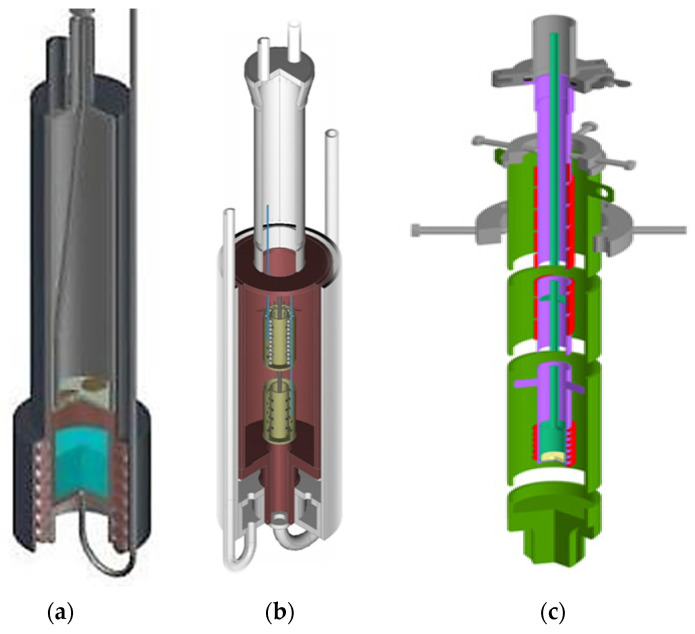
Irradiation ampoule devices for experiments: (**a**) in tritium and helium generation and release from lithium-containing materials (Li, Pb_83_Li_17_, lithium capillary-porous system); (**b**) in irradiation of lithium ceramics Li_2_TiO_3_; (**c**) in irradiation of two-phased lithium ceramics Li_2_TiO_3_-Li_4_SiO_4_.

The authors of the current paper have been conducting experimental studies on the neutron irradiation of various lithium ceramics and other lithium-containing materials at the WWR-K (Almaty, Kazakhstan) and the IVG1.M (Kurchatov, Kazakhstan) research reactors for many years [24,25]. The experiments were carried out both in the mode of purging samples with various gases and without it. Examples of typical irradiation devices for placing test samples inside the reactor core are shown in Figure 1 [26,27]. As shown by the thermophysical and neutronics calculations, as well as many years of experience in carrying out this type of work, it is important to take the thermophysical properties, shape and composition of a particular sample into account. 

It is important to note that in the absence of helium purging, the capsule with the sample, i.e., in the mode of vacuum extraction of gases released from the sample, it becomes possible to analyze the helium which is generated in the lithium-containing material under irradiation.

The purpose of this work was to simulate the heating of a mono-sized pebble bed (1 mm in diameter) of two-phase lithium ceramic 25 mol%Li_2_TiO_3_+75 mol%Li_4_SiO_4_ in an ampoule device during neutron irradiation at the WWR-K research reactor under vacuum conditions and to determine experimental parameters in order to prevent heating of the lithium ceramics up to the Li_4_SiO_4_-Li_2_SiO_3_ phase transition temperatures (>900 °C) [28,29]. Irradiation of ceramics in the absence of helium purging is caused by the need to use mass spectrometric analysis of gases formed in the sample (tritium, helium, etc.), which is carried out during high-vacuum pumping.

## 2. Calculation of the Effective Thermal Conductivity Coefficient of Lithium Ceramic Pebble Bed Depending on Helium Pressure for 2D and 3D Geometries

To assess the influence of helium pressure and the pebble-pebble contact area of lithium ceramics on the effective thermal conductivity of the pebble bed, model calculations were carried out using the COMSOL Multiphysics software package [30] and the parameters of the built-in materials: helium, lithium metatitanate and lithium orthosilicate.

**Figure 2:**
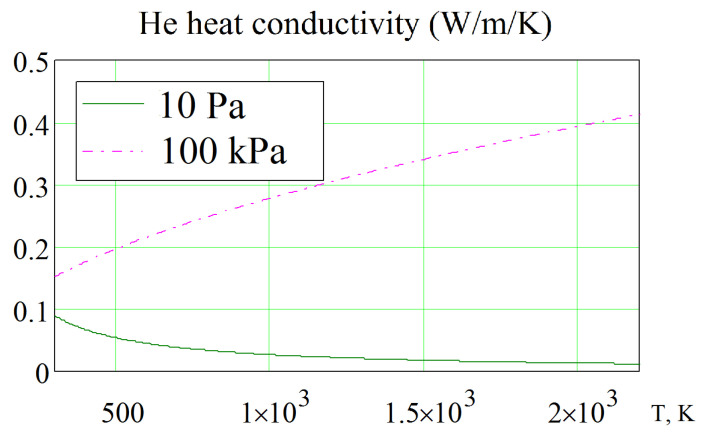
Thermal conductivity of helium at atmospheric pressure (100 kPa) and in a vacuum (10 Pa).

In this case, for irradiation of lithium ceramics in a vacuum, helium purging was not used. At low pressures (up to 10 Pa), helium is formed only as a result of ^6^Li(n,α)T nuclear reaction with the formation of tritium and helium. The thermal conductivity of such rarefied helium in the gaps between the pebbles of lithium ceramics and in its pores was described by the Knudsen flow. The dependence of the thermal conductivity on temperature used in calculations for a pressure of 10 Pa is shown in Figure 2 [31].

**Figure 3:**
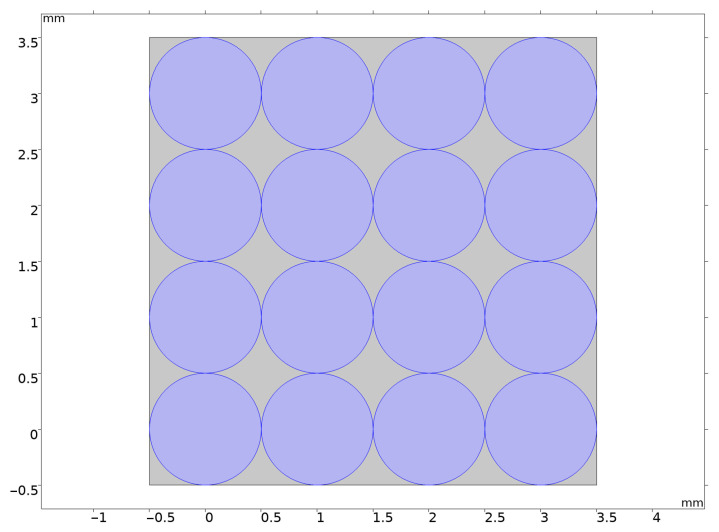
Two-dimensional model of 16 pebbles pebble bed made of two-phase lithium ceramics.

For a two-dimensional model of the pebble bed, a model of 16 two-phase lithium ceramic balls with a diameter of 1 mm in contact with each other was used as shown in Figure 3. The diameter of the ball-to-ball contact zones comprises about 10 μm. The gray areas in the figure are filled with helium.

The ceramic was described as a composite porous medium consisting of lithium orthosilicate (72%) and lithium metatitanate (23%) and 5% porosity. An incoming heat flux *Q* was applied to the upper boundary of the rectangular region. The sides of the rectangle were thermally insulated (there is no heat flux). On the lower edge, cooling was carried out by convection flow *q* according to the law:

(1) $$q=h\cdot (T_{ext}-T),$$

where *T_ext_* is the ambient temperature equal to 20 °C; *T* is the temperature at the lower boundary of the pebble bed, °C; *h* is heat transfer coefficient, W/(m^2^∙°C). 

The input flow *Q* and the heat transfer coefficient *h* were chosen arbitrarily so that the pebble bed temperature would not exceed 800 °C (the permissible value for heating lithium ceramics at which no phase changes occur in lithium orthosilicate). In this case, *Q* was equal to 7000 W/m^2^ and h = 10 W/m^2^/°C.

**Figure 4:**
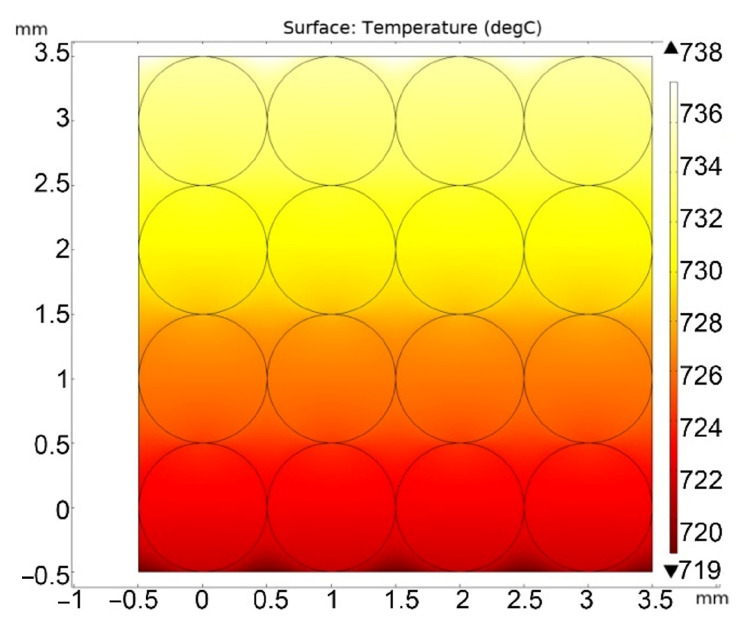
Stationary temperature distribution in the pebble bed at a helium pressure of 100 kPa.

**Figure 5:**
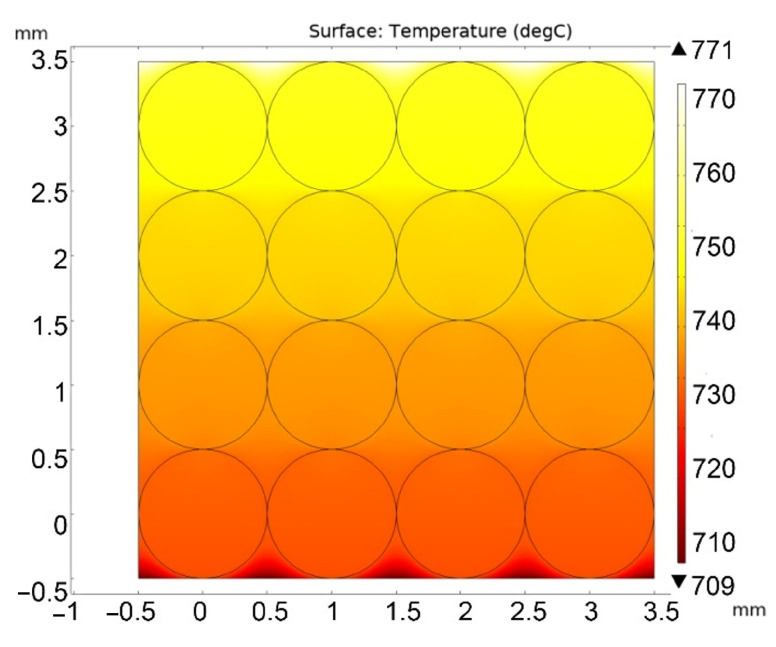
Stationary temperature distribution in the pebble bed at a helium pressure of 10 Pa.

Next, the calculation of the stationary regime of heat flow and temperature field over the pebble bed was carried out for atmospheric helium pressure and for a pressure of 10 Pa. Figure 4 and Figure 5 show solutions for the case of high and low helium pressure (100 kPa and 10 Pa), respectively.

Based on the calculation results, it is possible to determine the effective thermal conductivity of the lithium ceramics pebble bed using the formula:

(2) $$k_{eff}=Q\cdot \frac{L}{T_{max}-T_{min}},$$

where *Q* = 7000 W/m^2^; *L* = 4 mm (pebble bed height); $T_{max}$ and $T_{min}$—maximum and minimum temperatures over the entire pebble bed area, °C.

Thus, according to the calculation results, values were obtained for *k_eff_* = 1.47 W/m/°C (at 100 kPa) and *k_eff_* = 0.45 W/m/°C (at 10 Pa), respectively. That is, the absence of helium pressure led to a threefold decrease in the thermal conductivity of the two-dimensional pebble bed.

**Figure 6:**
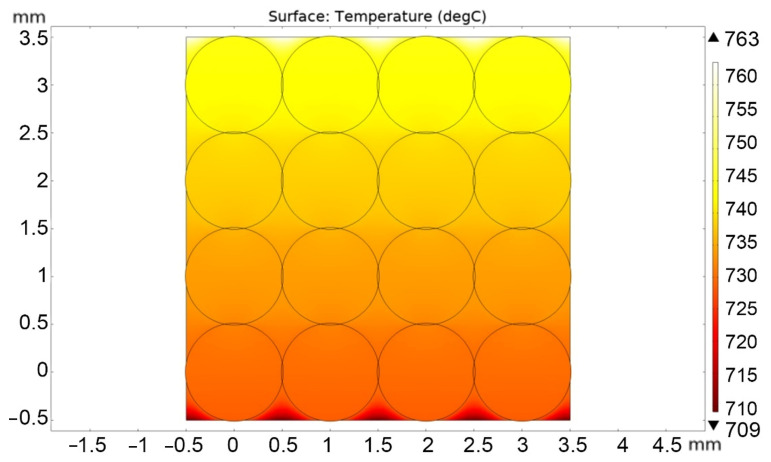
Stationary temperature distribution in the pebble bed at a helium pressure of 10 Pa and an increased thermal contact area.

Next, as an example, it was calculated how the thermal conductivity of the pebble bed would increase if it was “compressed” so that the diameter of the contact area of the pebbles increased to 100 μm. To do this, in the geometry of the model, the radii of the pebbles were increased to 0.505 mm, without changing the distance between them. As a result, the temperature distribution in the pebble bed at a helium pressure of 10 Pa and an increased thermal contact area was obtained, as shown in Figure 6. The effective thermal conductivity value turned out to be 1.55 W/m/°C for 100 kPa and 0.52 W/m/°C for 10 Pa, respectively.

**Figure 7:**
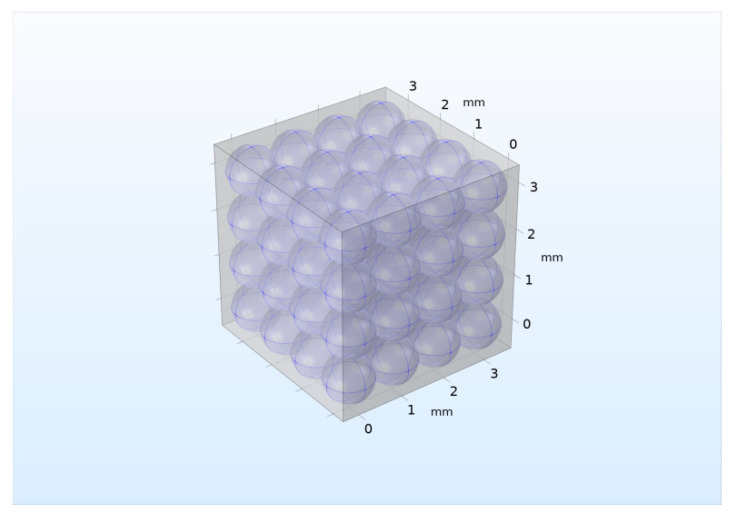
Three-dimensional model of pebble bed with 64 pebbles of two-phase lithium ceramic.

It is clear that the two-dimensional model, due to the requirement of infinity in the third spatial coordinate, provides only initial estimates of the effective heat transfer of a real three-dimensional and spatially limited pebble bed. Therefore, similar calculations were carried out for a three-dimensional pebble bed of 64 pebbles, shown in Figure 7. In Figure 7, gray areas are filled with helium.

**Figure 8:**
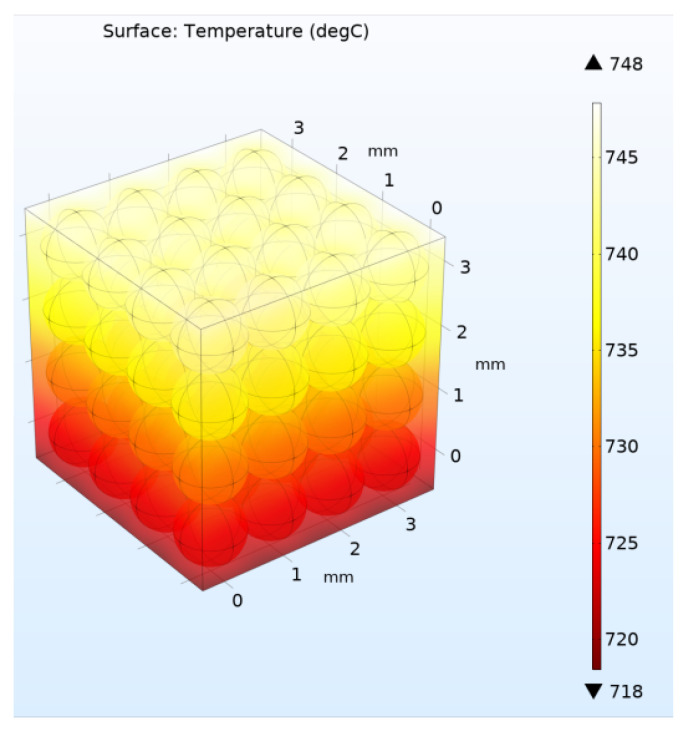
Stationary temperature distribution in the pebble bed at a helium pressure of 100 kPa in three-dimensional model with 64 pebbles.

**Figure 9:**
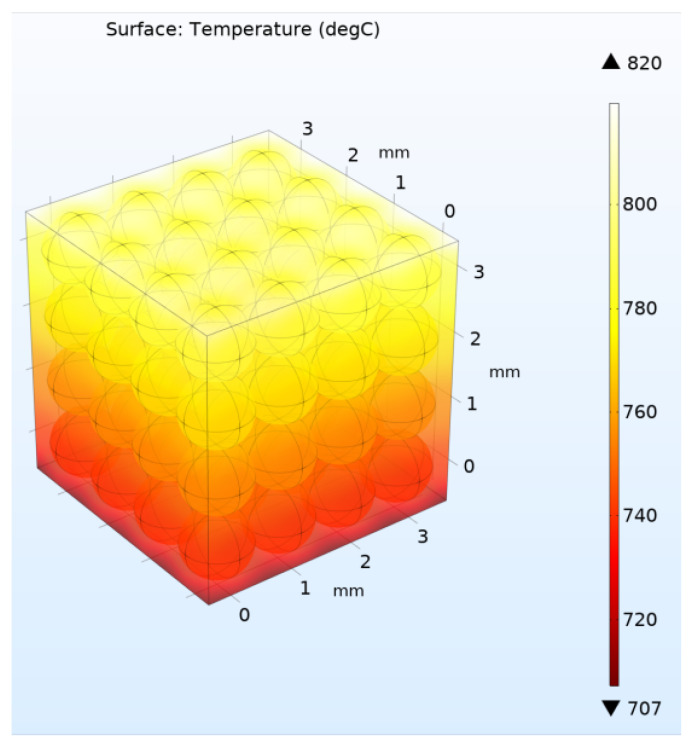
Stationary temperature distribution in the pebble bed at a helium pressure of 10 Pa in three-dimensional model with 64 pebbles.

Further, the calculation was carried out similarly to the above case of a two-dimensional model with the same heating and cooling flows *Q* and *q* at the upper and lower boundaries, respectively. The four lateral boundaries were assumed to be ideal heat insulators. Figure 8 and Figure 9 show the solutions for the three-dimensional model for the case of high and low helium pressure in the pebble bed (100 kPa and 10 Pa), respectively.

Thus, according to the calculation results, values were obtained for *k_eff_* = 0.93 W/m/°C (for 100 KPa) and *k_eff_* = 0.248 W/m/°C (for 10 Pa), respectively (Figure 8 and Figure 9). That is, the absence of helium pressure led to a decrease in the thermal conductivity of the three-dimensional pebble bed by almost four times.

**Figure 10:**
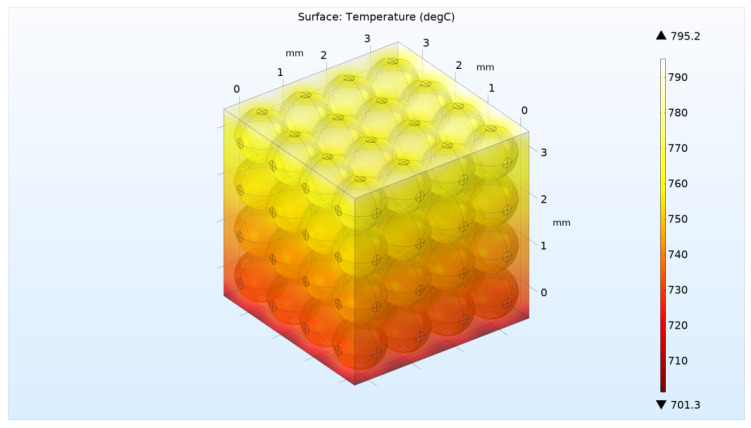
Stationary temperature distribution in the pebble bed at a helium pressure of 10 Pa and an increased thermal contact area in three-dimensional model with 64 pebbles.

Next, calculations were carried out for a tightly compressed pebble bed (Figure 10). As a result, an effective thermal conductivity value was obtained equal to *k_eff_* = 1.11 W/m/°C for a pressure of 100 kPa and *k_eff_* = 0.298 W/m/°C for a pressure of 10 Pa.

As an intermediate conclusion, it can be noted that the obtained values of the effective thermal conductivity of the filling of two-phase ceramic pebbles correspond to the literature data for real pebble beds [32,33,34], which confirms the correctness of the above calculations.

## 3. Modeling the Heating of a Real Irradiation Device Filled with Two-Phase Lithium Ceramics Pebble Bed during Neutron Irradiation in a Vacuum

The purpose of these calculations was to determine the stationary temperature distribution in the irradiation device and in the pebble bed of lithium ceramics in particular, under the influence of its own heat released in the lithium ceramics as a result of ^6^Li(n,α)T nuclear reaction. According to neutronics calculations carried out previously, it is necessary to load over 2 g of lithium ceramic pebbles into the ampoule device to ensure the release of nuclear reaction products in quantities sufficient for registration by a gas mass analyzer.

**Figure 11:**
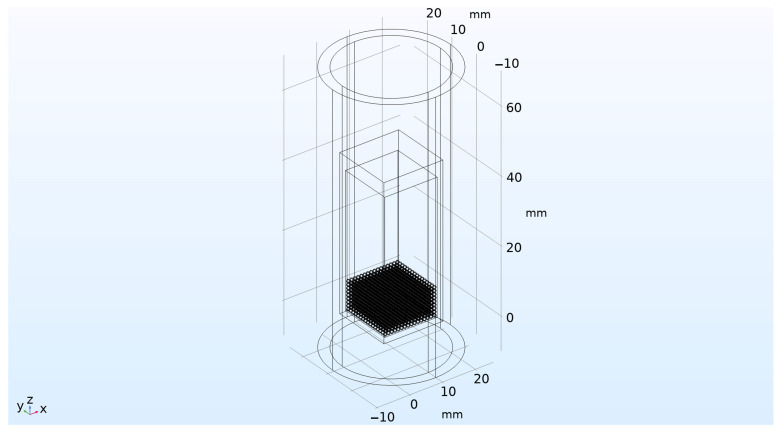
Calculation model of the device for neutron irradiation of a pebble bed of 2304 lithium ceramic pebbles in vacuum.

For calculations, a simplified model of an ampoule irradiation device was developed, taking into account the required parameters and physical mechanisms of the heat and mass transfer. The device diagram is shown in Figure 11.

**Figure 12:**
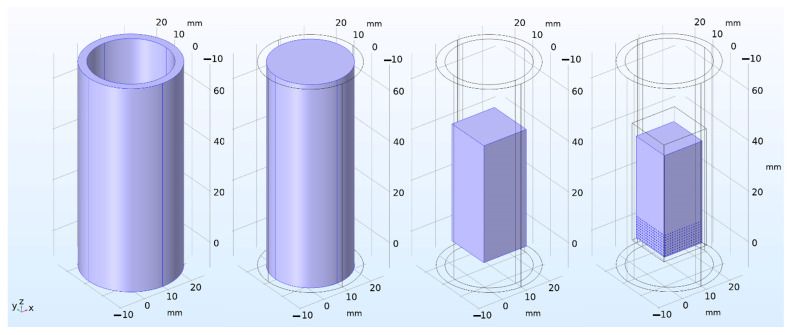
Main domains of the irradiation device model. From left to right: aluminum tube; purge gas—helium (at a pressure of 1 atm); stainless steel capsule; low-pressure helium (10 Pa) with lithium ceramic pebble bed.

The irradiation device was a case made of SS304 type stainless steel, with a wall thickness of 1 mm and an upper flange 5 mm thick (through which vacuum pumping of the device volume was ensured). The pebble bed of lithium ceramics was located at the bottom of the capsule in the form of an ordered array, with dimensions of 16 × 16 × 9 pebbles of 1 mm in diameter each. The volume of the capsule was evacuated (i.e., was under constant pumping) with a residual helium pressure in it equal to 10 Pa (this partial pressure of helium was supposed to be constant throughout the entire irradiation experiment). The capsule with irradiated ceramics was located in the internal volume of the irradiation device filled with helium at atmospheric pressure (100 kPa), which, together with the thermal radiation of the capsule, cooled it down. The body of the irradiation device was an aluminum tube with an outer diameter of 36 mm and a wall thickness of 3 mm. The temperature of outer surface of the aluminum pipe was 40 °C, which corresponded to the condition of its cooling with water from the cooling circuit of the WWR-K reactor. Figure 12 shows the main domains of the irradiation device model separately.

According to the task, all solid materials of the device emitted heat under reactor irradiation. Based on the results of neutron-physical calculations, the specific heat release of lithium ceramics was set to be 50.1 W/cm^3^, stainless steel—8.1 W/cm^3^ and aluminum—3.6 W/cm^3^.

The temperature of all components at the initial time was 40 °C. During the calculation, this temperature was maintained on the outer surface of the aluminum pipe. It is assumed that the purge helium moved in a laminar flow using a convection mechanism, under the influence of gravity and temperature differences in the cooling cavity. Low-pressure helium in the pebble bed area was considered as immobile.

The calculation was carried out in the Time-Dependent mode for the system reaching a stationary temperature distribution, with calculation of the helium movement in the cooling region. The calculation was made for a process duration of 750 s.

**Figure 13:**
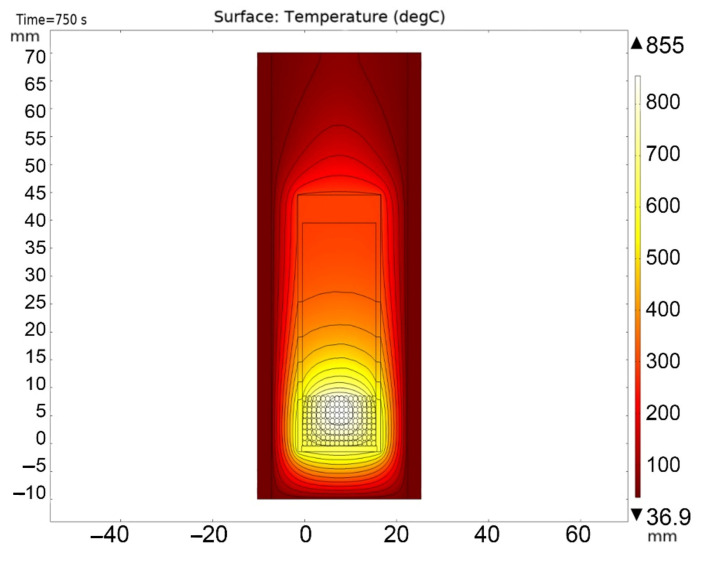
Final temperature distribution in the ampoule device and pebble bed at time 750 s at 10 Pa helium pressure.

**Figure 14:**
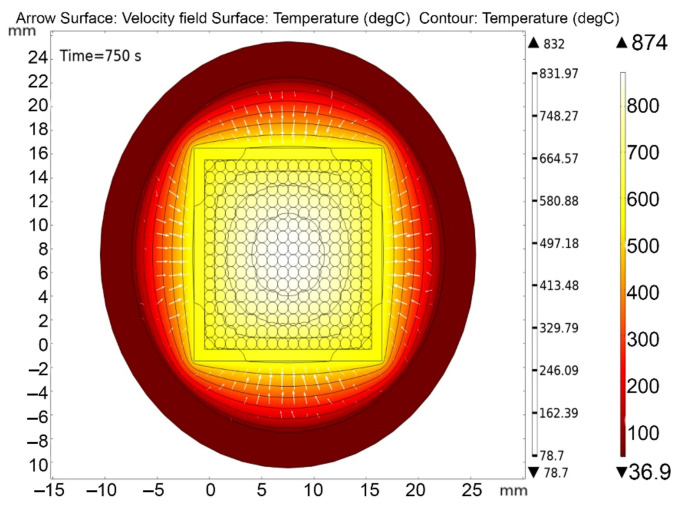
Final temperature distribution in the ampoule device and pebble bed at time 750 s (cross section through the 5-th layer of pebbles) at 10 Pa helium pressure.

**Figure 15:**
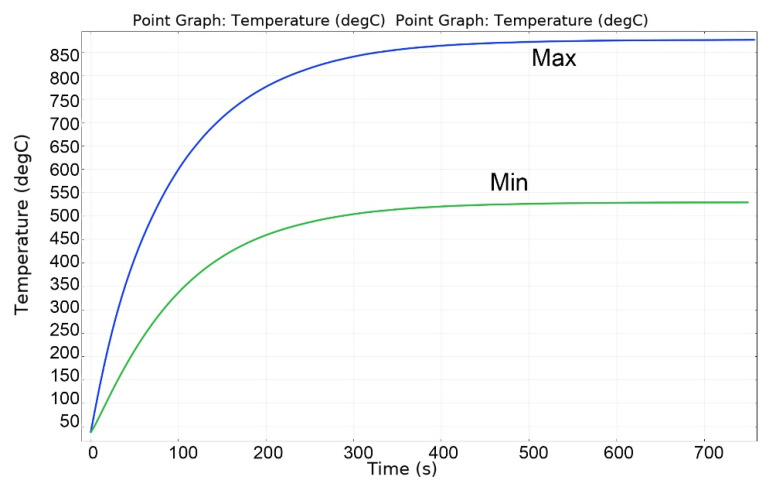
Changes in the maximum and minimum temperatures of ceramics during irradiation at 10 Pa of helium pressure in the pebble bed.

**Figure 16:**
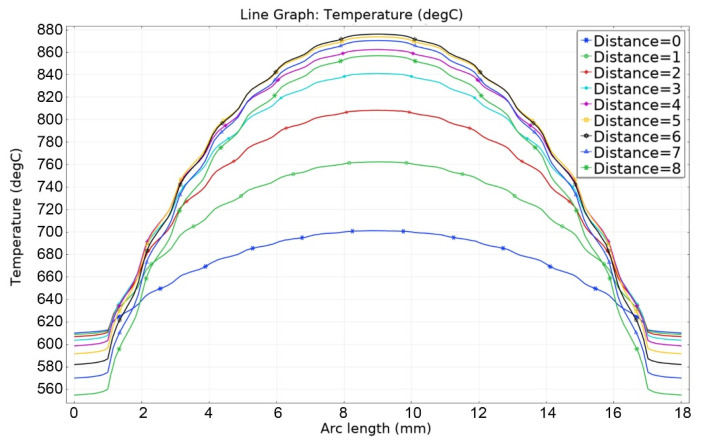
Temperature distribution in the layers of the pebble bed (0—bottom layer, 8—top) and across the capsule walls at 10 Pa of helium pressure.

**Figure 17:**
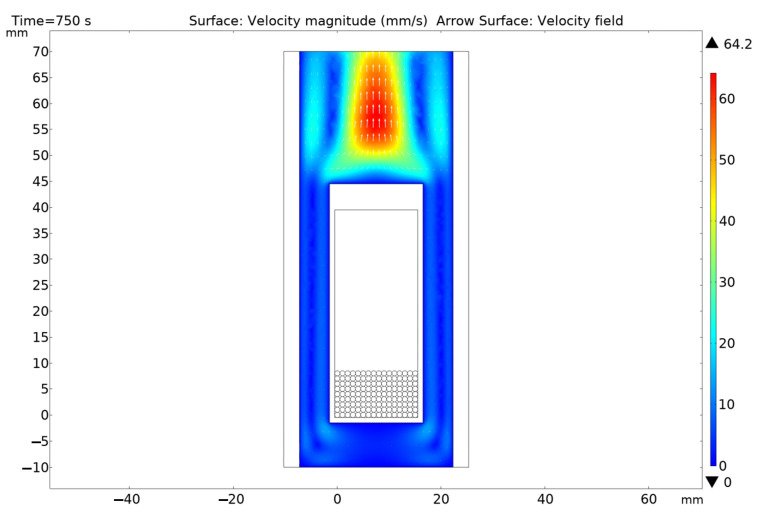
Velocity and directions of helium convection flows in the cooling region.

Figure 13, Figure 14, Figure 15, Figure 16 and Figure 17 show the main calculation results.

As can be seen in Figure 13, Figure 14, Figure 15, Figure 16 and Figure 17, at a helium pressure in the pebble bed of 10 Pa, the maximum temperature of some pebbles reached 875 °C. The temperature difference across the ceramic pebbles reached 350 °C, while approximately 20% of the pebbles in the center of the pebble bed heated up to temperatures close to the critical value of 820–870 °C. The temperature of the ceramics in contact with the capsule walls did not exceed 660 °C, and the temperature of the outer surface of the capsule does not exceed 610 °C.

This means that it is impossible to determine the maximum temperature of the ceramics in the capsule by measuring the temperature of its outer wall (according to thermocouple readings) without using the results of thermophysical calculations for each type of ceramics, its quantity, specific heat release and pebble size.

**Figure 18:**
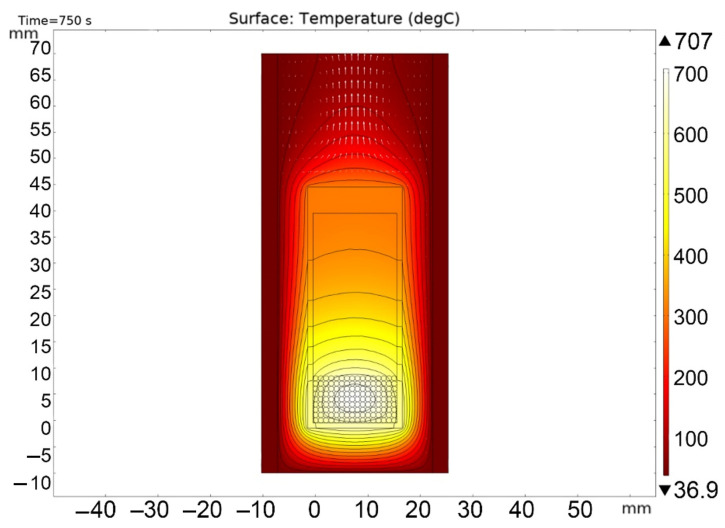
Final temperature distribution in the ampoule device and pebble bed at time 750 s at 100 kPa of helium pressure.

**Figure 19:**
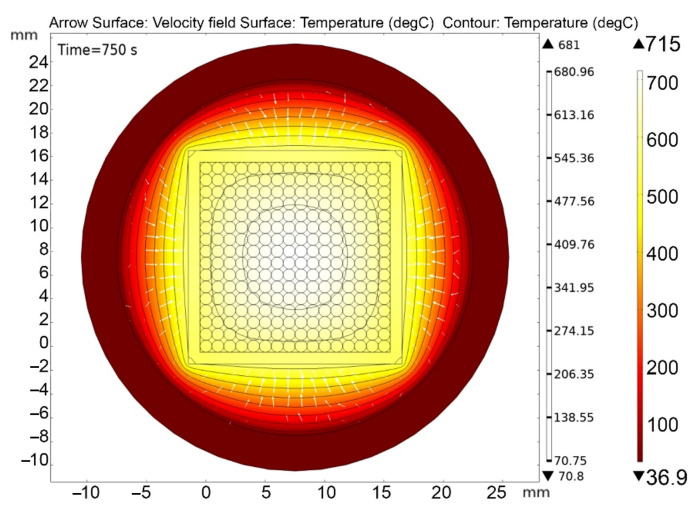
Final temperature distribution in the ampoule device and pebble bed at time 750 s (cross section trough the 5-th layer of pebbles) at 100 kPa of helium pressure.

**Figure 20:**
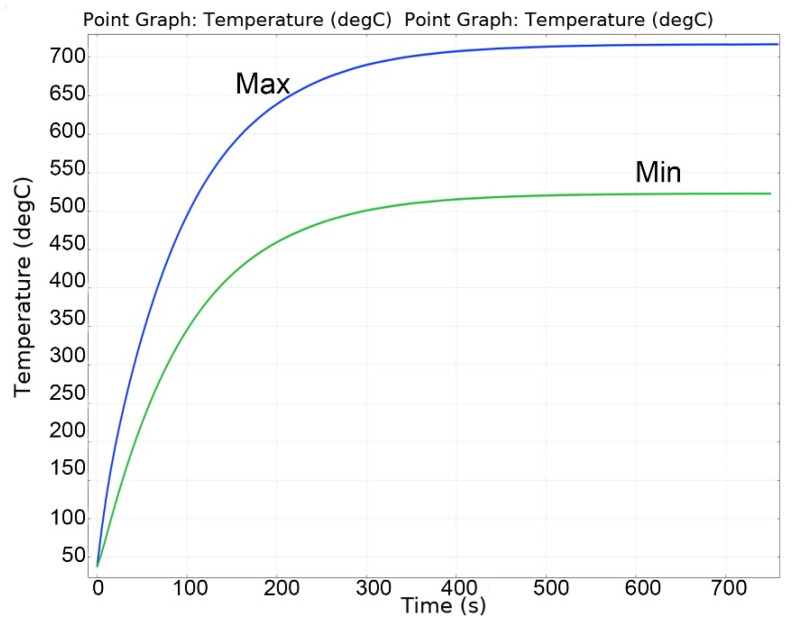
Dynamics of changes in the maximum and minimum temperatures of ceramics during irradiation at 100 kPa of helium pressure.

**Figure 21:**
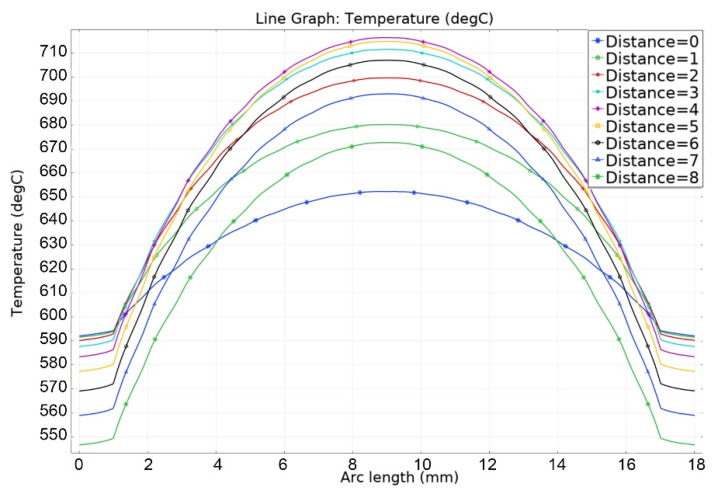
Temperature distribution in the layers of the pebble bed (0—bottom layer, 8—top) and along the capsule walls at 100 kPa of helium pressure.

For comparison, Figure 18, Figure 19, Figure 20 and Figure 21 show similar images obtained for the case of ceramic pebble bed with helium at atmospheric pressure (100 kPa).

Based on Figure 18, Figure 19, Figure 20 and Figure 21, it is clear that at a helium pressure in the pebble bed of 100 kPa, the maximum temperature of ceramics decreased significantly (~715 °C), the temperature difference across the ceramic pebbles reached 200 °C, while the temperature of the ceramics in contact with the capsule walls did not exceed 630 °C and the temperature of the capsule outer surface did not exceed 590 °C. That is, even in the case of helium pressure in the pebble bed of 100 kPa, the temperature of the ceramic exceeded the temperature of outer wall of the capsule by 100–120 °C.

## 4. Conclusions

The calculations show that the effective thermal conductivity of a single-sized pebble bed (1 mm in diameter) of two-phase lithium ceramic 25 mol%Li_2_TiO_3_+75 mol%Li_4_SiO_4_ in a vacuum (*k_eff_* = 0.3 W/m/°C) is an order of magnitude lower than the thermal conductivity of the Li_2_TiO_3_ (~2.5 W/m/°C) and Li_4_SiO_4_ (3.2 W/m/°C) ceramics themselves. 

The effective thermal conductivity of a pebble bed significantly decreased (four times) when it was irradiated with neutrons in a vacuum (at a helium pressure of approximately 10 Pa) compared to a similar calculation at pressure of helium of 100 kPa in the pebble bed.

As can be seen in the calculation results, with a specific heat release of 50 W/cm^3^ from the pebble bed and a helium pressure in the pebble bed of 10 Pa, the maximum temperature of some pebbles reached 875 °C. The temperature difference across the ceramic pebbles reached 350 °C, with approximately 20% of the pebbles in the center of the pebble bed heating up to temperatures close to the critical value of 870 °C, while the temperature of the ceramics in contact with the capsule walls did not exceed 660 °C, and the temperature of the capsule’s outer surface did not exceed 610 °C. This means that it is difficult to evaluate the maximum temperature of the ceramics in the capsule by measuring the temperature of its outer metal wall (according to the thermocouple readings) without using the results of the thermophysical calculations for each type of ceramic, taking its quantity, specific heat release and the pebble size(s) into account.

To control the temperature of the ceramics during an irradiation experiment in a vacuum, an in-capsule thermocouple should be used, placed in the center of the pebble bed. Measuring the temperature of the pebble bed based on the capsule wall temperature can lead to overheating of the ceramics and phase changes.

According to our calculations, the use of mechanical compression of the pebble bed, given a diameter of the contact area of 100 μm, leads to an increase in thermal conductivity of the pebble bed by ~10% (possibly with some unobvious losses in the mechanical strength of individual pebbles).
